# Met-CCL5 represents an immunotherapy strategy to ameliorate rabies virus infection

**DOI:** 10.1186/s12974-014-0146-y

**Published:** 2014-08-21

**Authors:** Ying Huang, Shaozhuo Jiao, Xiaoyan Tao, Qing Tang, Wentao Jiao, Jun Xiao, Xiaoyan Xu, Yanbo Zhang, Guodong Liang, Hongyan Wang

**Affiliations:** State Key Laboratory for Infectious Disease Prevention and Control, National Institute for Viral Disease Control and prevention, Chinese Center for Disease Control and Prevention, 155 Changbai Road, Beijing, 102206 Changping District China; State Key Laboratory of Cell Biology, Institute of Biochemistry and Cell Biology, Shanghai Institutes for Biological Sciences, Chinese Academy of Sciences, 320 Yueyang Road, Shanghai, 200031 China

## Abstract

**Background:**

Infection of rabies virus (RABV) causes central nervous system (CNS) dysfunction and results in high mortality in human and animals. However, it is still unclear whether and how CNS inflammation and immune response contribute to RABV infection.

**Methods:**

Suckling mice were intracerebrally infected with attenuated RABV aG and CTN strains, followed by examination of chemokine or cytokine production, inflammatory cell infiltration and neuron apoptosis in the brain. Furthermore, the suckling mice and adult mice that were intracerebrally infected with aG and the adult mice that were intramuscularly infected with street RABV HN10 were treated with CCL5 antagonist (Met-CCL5) daily beginning on day 2 postinfection. The survival rates and inflammation responses in the CNS of these mice were analyzed.

**Results:**

Excessive CCL5 in the CNS was associated with CNS dysfunction, inflammation, and macrophage or lymphocyte infiltration after attenuated or street RABV infection. Administration of exogenous CCL5 induced excessive infiltration of immune cells into the CNS and enhanced inflammatory chemokine and cytokine production. Met-CCL5 treatment significantly prolonged survival time of the suckling mice inoculated with aG and adult mice infected with aG and HN10.

**Conclusions:**

These results suggest that CCL5 in the CNS is a key regulator involved in inducing rabies encephalomyelitis. Furthermore, treatment with the CCL5 antagonist Met-CCL5 prolongs survival time of the mice infected with attenuated or street RABVs, which might represent a novel therapeutic strategy to ameliorate RABV infection.

## Background

Rabies virus (RABV) is a highly neurotropic virus that causes lethal central nervous system (CNS) disease in many species of mammals including humans [1]. Although rabies has been well controlled in the United States and other developed countries by vaccination in animals, it is still a public health threat, causing more than 55,000 human deaths worldwide each year [2]. Furthermore, no therapy has proved effective to cure rabid patients once rabies encephalitis develops or once the clinical symptoms appear.

Immune responses and CNS dysfunction are two main factors to be considered during RABV infection. Although RABV infection is invariably lethal in the absence of protective immune responses, several studies have argued that excessive immune responses may not always be beneficial for RABV infection. Attenuated RABV activates innate immune responses and induces extensive inflammation, apoptosis and neuronal degeneration in the CNS in experimental animals [3-6]. Moreover, the expression of the genes involved in innate immune and antiviral responses were highly upregulated after infection with attenuated RABV, especially those related to the alpha/beta interferon (IFN-α/β) signaling pathways, inflammatory cytokines and chemokines, including interleukin-6 (IL-6), IL-1α/β, IL-10, CXCL10/IP-10 and CCL5/RANTES [7-9]. However, it has been shown that overexpression of these chemokines (such as CXCL10 and CCL5) is closely correlated with severe enhancement of blood-brain barrier (BBB) permeability and excessive infiltration and accumulation of inflammatory cells in the CNS, which contributes to the increased pathogenicity in neurological diseases [10-12].

Most street RABVs evade the host innate immune system and fail to induce protective virus neutralizing antibody (VNA) responses [13-16]. However, in some murine or dog experimental models infected with street RABVs, T cell and mononuclear cell infiltration in the CNS have been observed together with severe encephalitis in the late stage of infection [16-18]. Although inflammatory response in the early stage of infection is important for clearance of RABV from the CNS [19], there is no evidence to suggest that severe inflammation in the late stage is beneficial to or impedes the development of the disease.

Chemokines have been originally identified as chemotactic and pro-adhesive cytokines by their interaction with G protein-coupled receptors. CCL5 (also termed as RANTES) is a β chemokine and induces leukocyte migration by binding to CCR1, CCR3 or CCR5 [20,21]. An elevated level of CCL5 has been associated with a variety of inflammatory disorders [22,23]. As one of the CCL5 receptors, CCR5 also has a significant role in various diseases, such as AIDS [24], arthritis [25], *Toxoplasma gondii* infection [26], West Nile virus infection [27] and respiratory virus infection [28]. Met-CCL5, an N-terminally modified human CCL5, has been previously shown to inhibit activity at two rodent chemokine receptors CCR1 and CCR5 [29]. Targeting CCL5 or CCR5 with antagonists may have potential therapeutic usage to alleviate symptoms of these diseases [30,31].

In this study, mice infected with attenuated RABVs developed excessive inflammation in the CNS. CCL5 was the highest virus-induced chemokine among 40 inflammatory cytokines and chemokines, which promoted migration of macrophages and T cells into the CNS. Excitingly, administration of the CCL5 antagonist, Met-CCL5, alleviated rabies clinical symptoms and prolonged the survival time of the sucking mice infected with attenuated RABV or adult mice infected with attenuated or street RABVs. Met-CCL5 treatment significantly reduced pro-inflammatory chemokine or cytokine production in the CNS after RABV infection. The findings suggest that Met-CCL5 might be used as a novel therapeutic reagent to prolong the survival time after RABV infection.

## Methods

### Animals and virus strains

Suckling mice (7-day-old) and adult mice (6-week-old) were purchased from the Institute of Laboratory Animal Medicine at the Chinese Academy of Medical Sciences (CAMS & PUMC, Beijing, China) and housed in the BSL-3 facility of the Veterinary Research Institute at the Academy of Military Medical Sciences. All procedures were conducted in accordance with the guidelines for the Medical Laboratory Animal (1998) from Ministry of Health, China. All animal experiments were carried out as approved by the Institutional Animal Care and Use Committee, Chinese CDC (permission number: 12-0121). Three strains of rabies viruses isolated in China were used in this study, including aG [32], CTN [33] and HN10 [34]. The background for isolation, hereditary traits and genomic information of these strains were well documented. The parental virus of the aG strain was isolated from the brain of a rabid dog in 1931, and it was attenuated to a fixed strain by 30 passages in rabbits, 55 passages in primary hamster kidney cells (PHKC), and several passages in guinea pigs and PHKC [32]. The aG strain was pathogenic for adult mice by intracerebral (i.c.) inoculation, but non-pathogenic through peripheral infection; it was used as a vaccine in 1981 in China [32]. The parental virus of the CTN strain was isolated from a rabid patient and attenuated by passaging in mice and human diploid cells. The pathogenic-related amino acid residue in position 333 of glycoprotein was mutated from arginine (R) to glutamine (Q). This CTN strain has been approved as a vaccine strain by the World Health Organization (WHO) since 1983 [33]. The HN10 strain was a street virus strain isolated from a rabid patient in China in 2006 [34].

### Virus infection and exogenous injection of CCL5 or Met-CCL5

Suckling mice were i.c. infected with aG or CTN. Adult mice were i.c. infected with aG or intramuscularly (i.m.) infected with HN10 (that is, the muscles of the right thigh) (5.6 × 10^3^ FFU, fluorescent focus forming unit; 25 μl in DMEM medium) (n = 10). To assess migration and apoptosis of immune cells infiltrating the CNS, suckling mice were i.c. injected with CCL5 (5 μg/ml, 25 μl), and the same volume of sterile PBS was used for mock controls. For Met-CCL5 treatment, suckling mice were intraperitoneally (i.p.) administrated daily with Met-CCL5 (100 μg/ml, 100 μl) from day 0 postinfection (p.i.) and adult mice were i.p. injected daily with high dose (200 μg/ml, 100 μl), low dose (20 μg/ml, 100 μl) of Met-CCL5, or the control reagent (random sequence of the amino acids from Met-CCL5; 200 μg/ml, 100 μl) beginning on day 2 p.i.. Recombinant carrier-free Met-CCL5 was purchased from R&D (R&D, Minneapolis, MN, USA). Met-CCL5 was derived from *E.coli* and contained 69aa from Ser24 to Ser91 with an N-terminal Met. The carrier-free control peptide (purity ≥95%) with the same length but random sequence of amino acids from Met-CCL5 was chemically synthesized by Hanhong Chemical Co. Ltd (Hanhong, Shanghai, China).

### Preparation of immune cells in spleen or central nervous system

Spleens from RABV-infected mice were homogenized and filtered through a 70-μm nylon cell strainer (Corning, Union City, CA, USA) to prepare single cell suspension. After red blood cells were removed with a lysing solution, cells were prepared for culturing or staining. To isolate immune cells infiltrating the CNS, mouse brains were removed and homogenized using digestion buffer (HBSS containing 0.05% collagenase IV and 10 μg/ml DNase I) and then filtered through a 70-μm nylon cell strainer (Corning, Union City, CA, USA). After digesting at room temperature for 20 minutes, the homogenates were allowed to settle vertically for another 20 minutes [35]. The clear supernatant was collected and centrifuged, and the single cell solution was prepared for analysis by flow cytometry thereafter.

### Flow cytometry

Antibodies (Abs) for flow cytometry were purchased from eBioscience (anti-B220, −CD3, −CD4, −CD11b, −F480, and − CD8), BD Biosciences (anti-CCR5, −Annexin V, and -BrdU), and Cell Signaling Technology (anti-Caspase-3 Asp175). Staining processes were performed according to the manufacturer’s instruction. Briefly, for cell surface straining, cells were blocked with Fc γ MAb (0.5 μg/ml) for 30 min at 4°C. After being washed with PBS, cells were stained with antibodies against B220, CD3, CD4, CD11b, F480, CD8 or CCR5 for 30 min on ice with gentle shaking. For cell apoptosis analysis, fresh cells were stained with anti-Annexin V antibody and propidium iodide (PI), while pre-fixed and permeated cells were stained using anti-caspase-3 antibody. For BrdU incorporation assay, the RABV-infected mice (n ≥3) were i.p. injected with BrdU (10 mg/ml, 100 μl) for 24 hours (hrs); cells from brains were then collected. After being stained with cell surface markers for 30 min on ice, the cells were stained with anti-BrdU antibody according to the manufacturer’s instruction. Samples were processed using FACSCalibur or Accuri C6 (BD Biosciences, San Jose, CA, USA), and data were analyzed with the FlowJo software (Tree Star, Ashland, OR, USA).

### Western blot

Western blot was performed using the following primary antibodies: phospho-Akt (Ser 473) and phospho-Fak (Tyr 925) (Cell Signaling Technology, Danvers, MA, USA). Briefly, splenocytes from the RABV-infected and mock-infected mice were harvested, and splenocytes from the mock-infected mice were stimulated with or without 60 ng/ml CCL5 at 37°C for 5 min. After being washed with PBS, the cells were lysed using SDS-loading buffer and boiled for 10 min. Lysates were separated by sodium dodecyl sulfate polyacrylamide gel electrophoresis (SDS-PAGE) and transferred thereafter to a nitrocellulose membrane. After being blocked in TBST with 5% BSA for 1 hour (h), the membrane was incubated with primary antibodies overnight at 4°C, followed by incubation with horseradish peroxidase-labeled secondary antibody. Detection was performed with ECL substrate (Thermo Scientific, West Palm Beach, FL, USA).

### Inflammation antibody array assay

Mouse inflammation antibody array G (RayBiotech, Norcross, GA, USA) was used to determine the protein expression levels of various cytokines and chemokines in the brain samples. Briefly, proteins were extracted from brains of the moribund mice infected with aG, CTN or HN10 (n ≥3 in each group). The array was inoculated with 50-μg proteins in 100 μl of the supplied buffer and then treated with biotin-conjugated antibodies for 2 h. After being washed, the array was incubated with Alexa Flour 555-conjugated streptavidin for 2 h at room temperature, and the images were visualized using an Axon GenePix 4300A laser scanner (Molecular Devices, Sunnyvale, CA, USA).

### Quantitative real-time PCR

The relative mRNA expression levels of IL-1β, IL-6, IL-12, IL-17, CCL3, CCL5 and virus nucleoprotein were measured by quantitative real-time PCR (qRT-PCR). Briefly, total RNA was isolated from brains of the RABV-infected mice using TRIzol reagent (Invitrogen, Grand Island, CA, USA). cDNAs were synthesized from mRNA by Ready-To-Go You-Prime First-Strand Beads (Amersham Biosciences, Piscataway, NJ, USA) using d(N)6 as primers. qRT-PCR was performed using SYBR Green real-time PCR master mix on the CFX96 system (Bio-Rad, Hercules, CA, USA). To examine virus replication in brains, the mRNA levels of virus nucleoprotein were measured by qRT-PCR using specific primers to detect N protein. The mRNA copy numbers were normalized to the housekeeping gene β-actin.

### Preparation of bone marrow-derived macrophages

Bone marrow-derived macrophages (BMM) were generated using L929-cell conditioned medium (LCCM) as a source of granulocyte/macrophage colony-stimulating factor. Briefly, bone marrows were removed from tibias and femur bones of 8-week-old mice. Following red blood cell lysis and washing, cells were plated in DMEM medium (Invitrogen, Grand Island, CA, USA) supplemented with 10% fetal calf serum (CSF), 1% streptomycin/penicillin, and 30% LCCM. On day 7, BMMs were treated for 6 hrs with CCL5 (60 ng/ml), poly (I:C) (polyinosinic-polycytidylic acid), or poly (I:C) and CCL5 with or without Met-CCL5. The relative mRNA levels of cytokines or chemokines were evaluated by qRT-PCR.

### Transwell migration assay

Splenocytes were isolated from the RABV-infected mice and their migration ability with or without CCL5 stimulation was subjected to a transwell migration assay. Briefly, 600 μl RPMI1640 medium with or without 100 ng/ml CCL5 was added into a 24-well plate, and then splenocytes (0.5million/100 μl) were placed in the transwell inserts (Corning, Union City, CA, USA). The transparent polyester membranes of the inserts were immersed in the RPMI medium. After being incubated at 37°C for 6 hrs, the cells that had migrated through the polyester membrane into the RPMI medium were collected and counted under an Olympus CK40 light microscope (Olympus, Tokyo, Japan).

### Preparation and staining of brain section

RABV-infected mice were euthanized and perfused with PBS followed by 4% paraformaldehyde fixation. The brains were removed and fixed in 4% paraformaldehyde at 4°C for 24 hrs and then immersed in a 10% sucrose solution at 4°C for 48 hrs. Coronal frozen sections of brain tissue (20 μm) were cut on a Leica CM1900 microtome (Leica, Wetzlar, Germany). Sections were stained with cresyl violet to examine the pathological changes of neurons. To detect activated caspase-3, brain sections were incubated with PBS/0.2% Triton for 5 min, and then blocked with PBS containing 5% donkey serum and 0.1% Tween 20 for 2 hrs. They were then stained with rabbit anti-active caspase-3 polyclonal antibody (Promega, Madison, WI, USA) overnight at 4°C. After being washed, brain sections were incubated with Cy3-donkey anti-rabbit IgG antibody (Jackson Laboratories, West Grove, PA, USA) for 2 hrs at room temperature. For terminal deoxynucleotidyl transferase dUTP nick end labeling (TUNEL) staining, the frozen tissue sections were stained according to the manufacture’s protocols for DeadEnd Colorimetric TUNEL System kit (Promega, WI, USA). The average number of apoptotic cells in different regions of hippocampus (CA1, CA3 and DG) was quantified. To detect immune cell infiltration in the brain, T lymphocytes, macrophages, and activated microglia were stained with Alexa Fluor 488-anti-mouse CD3 monoclonal antibody (17A2), anti-mouse integrin αM/CD11b antibody, and Northernlights NL557 fluorescent secondary antibody (R&D, Minneapolis, MN, USA), respectively. The numbers of infiltrated CD3^+^ T cells in the brain were counted under 10× magnification for each slide. Images were taken from an Olympus FV500 confocal microscope (Olympus, Tokyo, Japan).

### Statistical analysis

Statistical significances between different groups were analyzed using a two-tailed Student’s t-test or two-way ANOVA, and statistical significance of survival rates was determined by the log rank test and Kaplan-Meier survival analysis. *P* <0.05 was considered statistically significant (*P <0.05; **P <0.01; ***P <0.001).

## Results

### Rabies virus infection induces high mortality and excessive inflammation in the central nervous system of suckling mice

**Figure 1:**
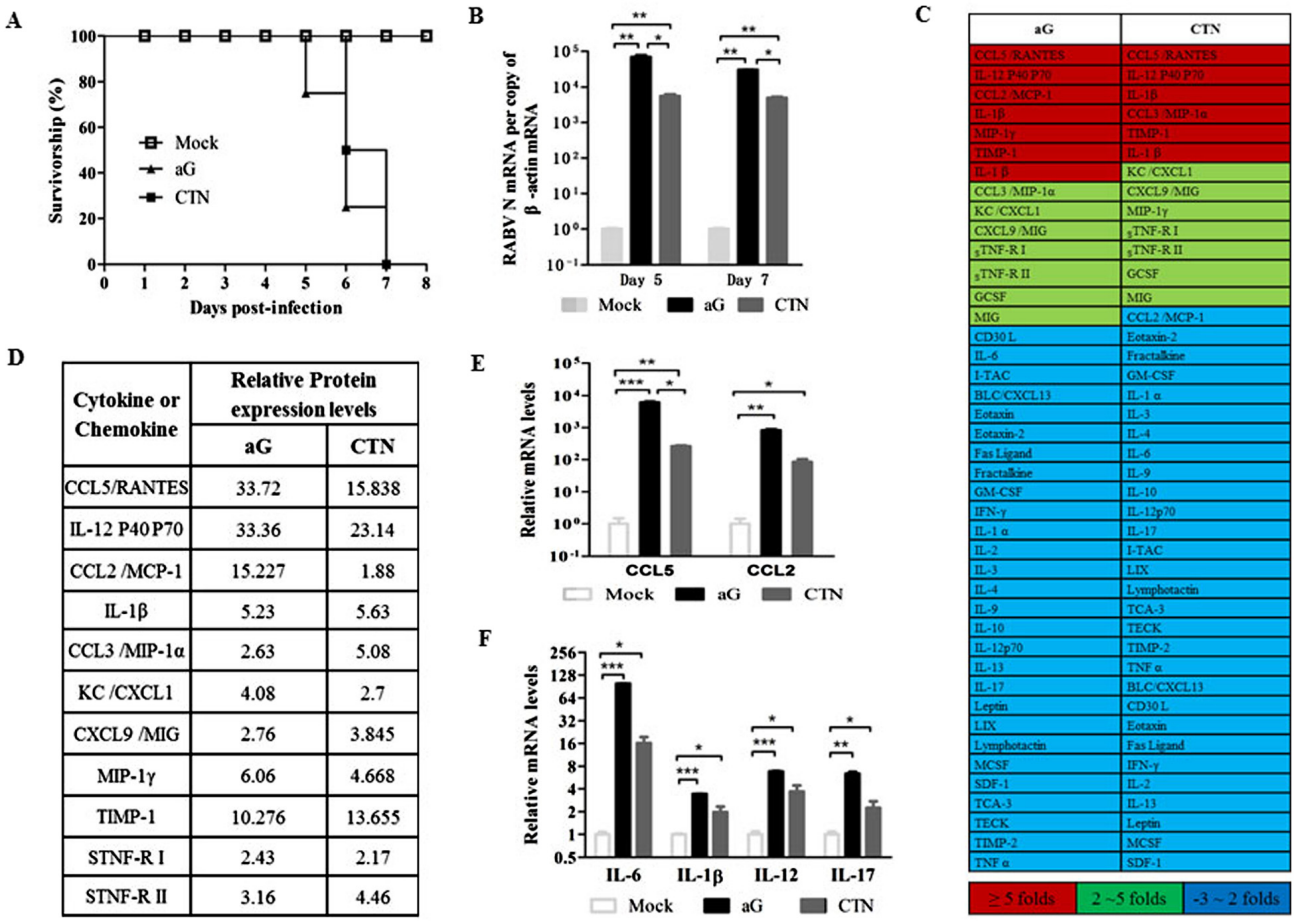
**Rabies virus (RABV) infection induces high mortality and excessive inflammation in the central nervous system of suckling mice.** Suckling mice were intracerebrally (i.c.) infected with aG or CTN (5.6 × 10^3^ FFU), and **(A)** the survival rates were monitored (n = 10 in each group). **(B)** The relative mRNA levels of RABV N protein were determined in aG- and CTN-infected mice on day 5 and day 7 postinfection (p.i.). **(C)** On day 7 p.i., the expression profiles of various inflammatory proteins in brain samples were evaluated by RayBio™ Mouse Inflammation Antibody Array G. **(D)** The protein levels of inflammatory molecules that were enhanced over 1.5-fold in the brain samples infected with aG and CTN are listed (n = 3). The relative mRNA levels of CCL5, CCL2 **(E)**, IL-6, IL-1β, IL-12, IL-17 **(F)** in brain samples were verified by quantitative reverse transcriptase PCR (qRT-PCR) after aG and CTN infection. Data were presented as means ± SD (*P <0.05; **P <0.01; ***P <0.001).

To identify the key immune effectors that contribute to the development of CNS inflammation and lethal RABV infection, suckling mice were i.c. infected with attenuated RABV strains, including CTN and aG. All the mice developed neurological symptoms, such as agitation, fury, and paralysis of hind limbs within a week p.i.. As shown in Figure 1A, 80% and 50% of suckling mice were dead at day 6 p.i. with aG and CTN, respectively. Also, 1 log more RABV N mRNA was detected in aG-infected mice than in CTN-infected mice (Figure 1B). To investigate whether the pathogenicity of these attenuated RABVs was related to overreaction of the host immune system and extensive inflammatory response in the CNS, RayBio™ Mouse Inflammation Antibody Array G was used to screen the protein expression levels of more than 40 inflammatory molecules, including cytokines, chemokines and growth factors. The inflammatory molecules that upregulated in varying levels in aG- or CTN-infected brains were listed in Figure 1C. There were 11 cytokines or chemokines that were enhanced at both the onset and late stages of RABV infection, and CCL5 was expressed with the highest level being 33.7 and 15.8 in aG- and CTN-infected mice, respectively (Figure 1D). Furthermore, the antibody array was randomly confirmed by qRT-PCR, and the results showed that pro-inflammatory chemokines including CCL5 and CCL2 (Figure 1E) and cytokines including IL-6, IL-1β, IL-12, and IL-17 (Figure 1F) were significantly enhanced after infection with aG and CTN. A previous microarray screening analysis on attenuated RABV infected mice was based on 22,626 mouse genes and showed that except for the induction of genes in the IFN-α/β signaling pathway, genes encoding inflammatory cytokines and chemokines were highly upregulated including CCL5 [6]. Another study, which used the Milliplex MAP 30-plex premixed mouse cytokine/chemokine magnetic bead panel, also proved that CCL5 was highly upregulated in attenuated rabies virus-infected mice [18]. Together, our and others’ studies suggested that attenuated RABV-infected mice induced a strong inflammatory immune reaction in the CNS.

### Attenuated rabies virus infection increases infiltration of immune cells into the central nervous system

**Figure 2:**
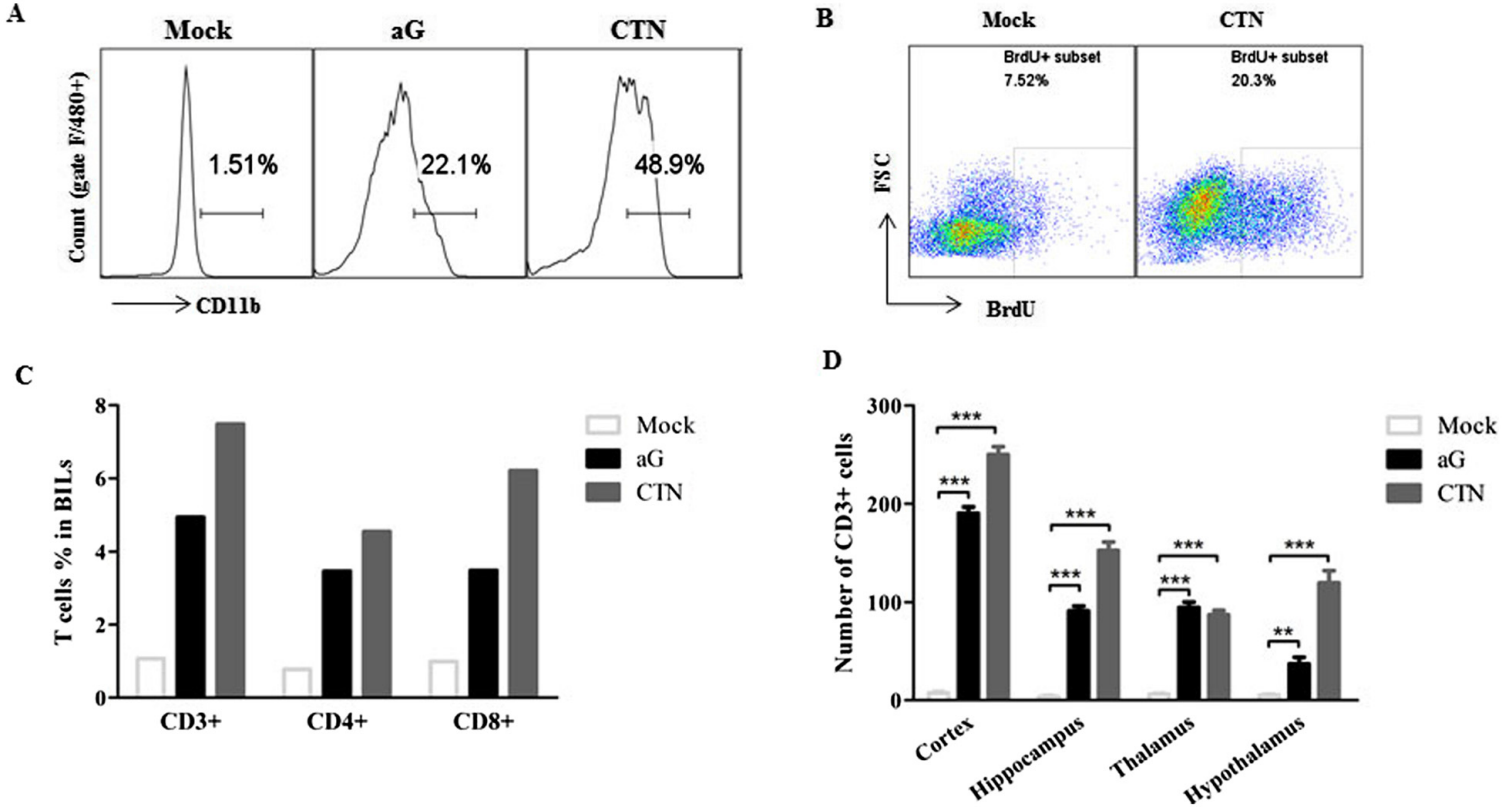
**Attenuated rabies virus (RABV) infection increases infiltration of immune cells into the central nervous system (CNS).** Suckling mice were intracerebrally (i.c.) infected with aG and CTN, and brains and spleens were harvested on day 7 postinfection (p.i.) (n ≥3, mean ± SD). The percentages of CD11b^hi^ F4/80^+^ cells **(A)**, CD3^+^, CD4^+^ and CD8^+^ T lymphocytes **(C)** from brain infiltration of lymphocytes (BILs) were measured by flow cytometry. **(B)** Suckling mice were i.c. infected with CTN (5.6 × 10^3^ FFU) or PBS and then intraperitoneally (i.p.) injected with BrdU on day 5 p.i.. BILs in the brain were isolated and subjected to flow cytometry analysis. **(D)** Number of CD3^+^ T cells infiltrated into different regions of the brain, including cerebral cortex, hippocampus, thalamus and hypothalamus, were quantified by immunohistochemistry (IHC) staining. Data were presented as means ± SD (**P <0.01; ***P <0.001).

To investigate whether excessive chemokines in the CNS affected recruitment of macrophages and T lymphocytes during RABV infection, brain infiltration of lymphocytes (BILs) were isolated and analyzed by flow cytometry. The infiltrated macrophages and activated resident microglia had been suggested to provide the first line of defense against infection in the CNS, which were identified as CD11b^hi^F4/80^+^ population as previously described [11,36]. As shown in Figure 2A, the percentages of infiltrated macrophages and activated microglia were significantly increased in aG- or CTN-infected mice when compared to those in mock-infected mice; the *in vivo* BrdU incorporation assay also showed that CD11b^hi^F4/80^+^ BILs were highly proliferated in mice infected with CTN (Figure 2B). This demonstrated that macrophages infiltrated the CNS, and resident microglia were activated in response to the attenuated RABVs infection. Similarly, CD3^+^, CD4^+^ and CD8^+^ T cells infiltrated the brains examined by flow cytometry (Figure 2C). This was confirmed by immunohistochemistry (IHC) staining of the infected brain tissue sections using anti-CD3 antibodies (Figure 2D), suggesting that T lymphocytes also infiltrated the CNS after attenuated RABV infection.

### CCL5/CCR5 is essential for mediating immune cell infiltration in the central nervous system of rabies virus-infected mice

**Figure 3:**
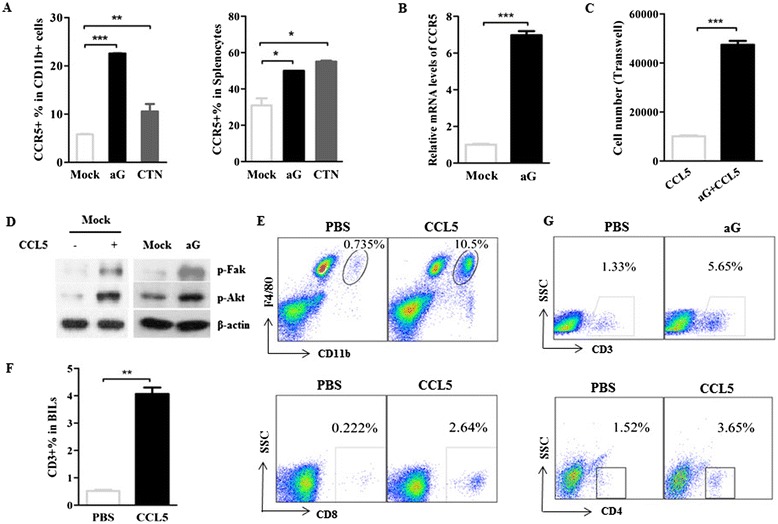
**CCL5/CCR5 is essential to mediate immune cell infiltration in the central nervous system (CNS) of aG- or CTN-infected mice. (A)** The percentages of CCR5^+^ cells in CD11b^+^ macrophages from brain infiltration of lymphocytes (BILs) or splenocytes in mice infected with rabies viruses (RABVs) were assessed using flow cytometry. **(B)** The relative mRNA level of CCR5 was analyzed in mock- or aG-infected brain samples on day 5 postinfection (p.i.). **(C)** The number of migrated splenocytes in response to CCL5 (100 ng/ml) was analyzed using *in vitro* transwell assay. **(D)** The phosphorylation of Fak and Akt in splenocytes from mock- or aG-infected mice was determined by western blot, while exogenous CCL5-stimulated splenocytes were used as a positive control. **(E and F)** Suckling mice were intracerebrally (i.c.) injected with exogenous CCL5 (25 μl, 5 μg/ml). On day 1 p.i., cells isolated from brains were collected to assess the percentages of F4/80^+^CD11b^hi^ microglia/macrophages, CD3^+^, CD4^+^ and CD8^+^ T lymphocytes using flow cytometry. **(G)** The percentage of infiltrated CD3^+^ T cells in the brain of mock- or CTN-infected mice was analyzed using flow cytometry.

CCL5/CCR5 has been well demonstrated to activate several types of immune cells and guide them to specific homing into target organs in some diseases [37,38]. Therefore, further investigation needs to determine whether CCL5/CCR5 mediates migration of the immune cells into the CNS during RABV infection. The increase in expression levels of surface CCR5 in macrophages or splenocytes in the aG- or CTN-infected mice was 1.5 to 5-fold that observed in the mock-infected mice (Figure 3A). The mRNA levels of CCR5 in the CNS were also upregulated in aG-infected mice compared to those levels in mock-infected mice (Figure 3B). Next, the chemotactic activity of splenocytes isolated from RABV-infected mice was assessed in response to CCL5 stimulation by Transwell™ assay *in vitro*. Consistent with the CCR5 expression enhancement, splenocytes isolated from aG-infected mice migrated more efficiently through the transparent polyester membrane in response to CCL5 stimulation (Figure 3C), which indicated that CCL5/CCR5 interaction promoted immune cell migration during RABV infection. Furthermore, the phosphorylation levels of Fak and Akt, two critical effectors for cell migration, were analyzed. After CCL5 stimulation, splenocytes isolated from mock-infected mice increased the phosphorylation levels of Fak and Akt (Figure 3D, left panel), which is in agreement with a previous study [39]. Interestingly, the phosphorylation levels of Fak and Akt were significantly enhanced in splenocytes after aG infection (Figure 3D, right panel). Together, these data suggest that CCR5 expression levels in immune cells increased after RABV infection, which induce Fak and Akt phosphorylation to promote cell migration in response to enhanced CCL5 in the CNS.

To further confirm that CCL5 was one critical immune regulator recruiting macrophages or T cells into brains, exogenous CCL5 was i.c. injected into mice. Resting microglia were identified as the CD11b^int^F4/80^+^ population, and the infiltrated macrophages and activated microglia were labeled as the CD11b^hi^F4/80^+^ population. As shown in Figure 3E, resting microglia existed in mice treated with either PBS or exogenous CCL5, whereas CD11b^hi^F4/80^+^ macrophages and activated microglia were significantly higher in the mice treated with exogenous CCL5. This suggests that exogenous CCL5 in the brain activates resting microglia or recruits macrophages into the CNS. Moreover, the percentages of CD3^+^, CD4^+^ and CD8^+^ T cells increased substantially more in mice treated with CCL5 than in those treated with PBS (Figure 3F). This result was consistent with the enhanced infiltration of CD3^+^ T cells into the CNS after RABV infection (Figure 3G).

### Exogenous CCL5 treatment and rabies virus infection enhance neuron apoptosis in the central nervous system

**Figure 4:**
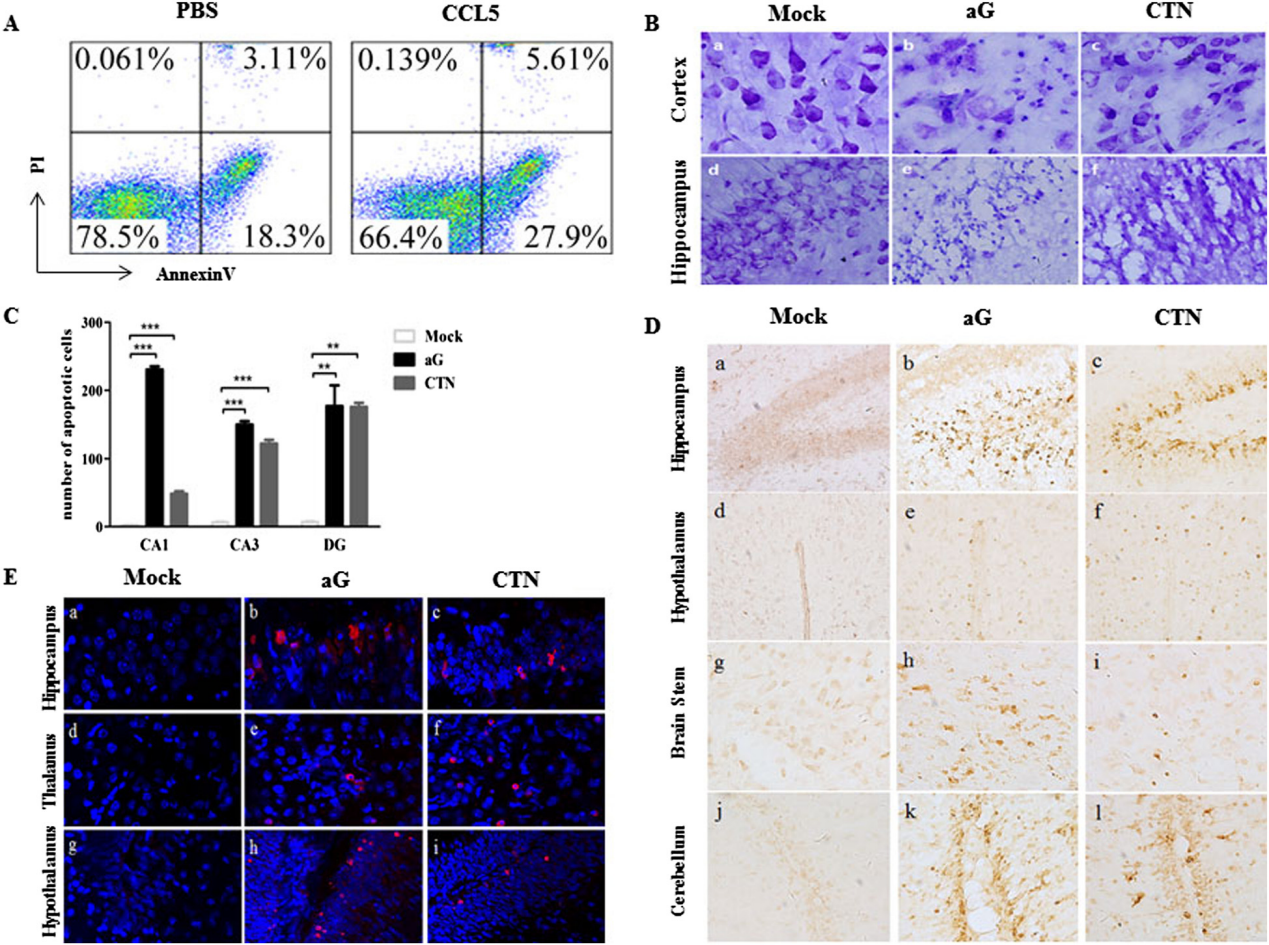
**Exogenous CCL5 treatment and rabies virus infection enhance neuron apoptosis in the central nervous system.** Mice were intracerebrally (i.c.) infected with PBS or exogenous CCL5 (25 μl, 5 μg/ml). **(A)** On day 1 postinfection (p.i.), single cell suspension was prepared from the brains and stained with propidium iodide (PI) and AnnexinV to analyze cell apoptosis by flow cytometry. **(B)** Suckling mice infected with aG and CTN (5.6 × 10^3^ FFU) were euthanized, and brains were removed to prepared frozen section on day 5 p.i.. Pathological changes of neurons, including irregular nuclear chromatin condensation, vacuolation, and neuron necrosis in cerebral cortex and hippocampus, were observed by cresyl violet staining (Magnification: 40×). **(C and D)** Brain sections were subjected to terminal deoxynucleotidyl transferase dUTP nick end labeling (TUNEL) staining, and the TUNEL-staining positive cells were quantified (n = 3). **(E)** Cleaved caspase-3 was stained with caspase-3 antibody (red), and the cell nuclei were stained with DAPI (blue). Image magnification: a-f (60×), g-i (20×). Data were presented as means ± SD (**P <0.01; ***P <0.001).

It was previously reported that encephalitis induced by attenuated RABVs could induce neuron damage or apoptosis, which plays an important role in the pathogenesis of RABV infection [5]. Since neurons, astrocytes and oligodendroglia in the CNS have limited capacity for self-renewal, fatal outcome or dysfunction may be shown when these cells are severely damaged after RABV infection. As shown in Figure 4A, the percentages of early apoptosis (AnnexinV^+^PI^−^, 27.9% versus18.3%) and late apoptosis/necrosis (AnnexinV^+^PI^+^, 5.61%versus 3.11%) of neurons increased more in mice injected with CCL5 than in those injected with PBS. Next, the degree of neuron damage after aG and CTN infection was further assessed. Mouse brain frozen sections were stained with cresyl violet, TUNEL and activated caspase-3 to analyze cytopathology and cell apoptosis after aG and CTN infection. Compared to mock-infected mice (Figure 4B, a/d), more severe neuronal cytopathology in the cerebral cortex and hippocampus were observed in aG- and CTN-infected mice (Figure 4B, b/e and c/f), characterized by irregular nuclear chromatin condensation, vacuolation and loss of neurons. Moreover, significantly more apoptotic DNA fragments were observed in the hippocampus (Figure 4C), hypothalamus, cerebellum and brain stem (Figure 4D) in aG- and CTN-infected mice than in mock-infected mice. In addition, the enhanced amount of cleaved/activated caspase 3 was observed in the CNS in aG- and CTN-infected mice (Figure 4E). These results suggest that CCL5 plays a critical role in inducing neuron apoptosis in the CNS in response to the attenuated RABV infection.

### Met-CCL5 treatment reduces central nervous system inflammation and prolongs survival time after rabies virus infection

**Figure 5:**
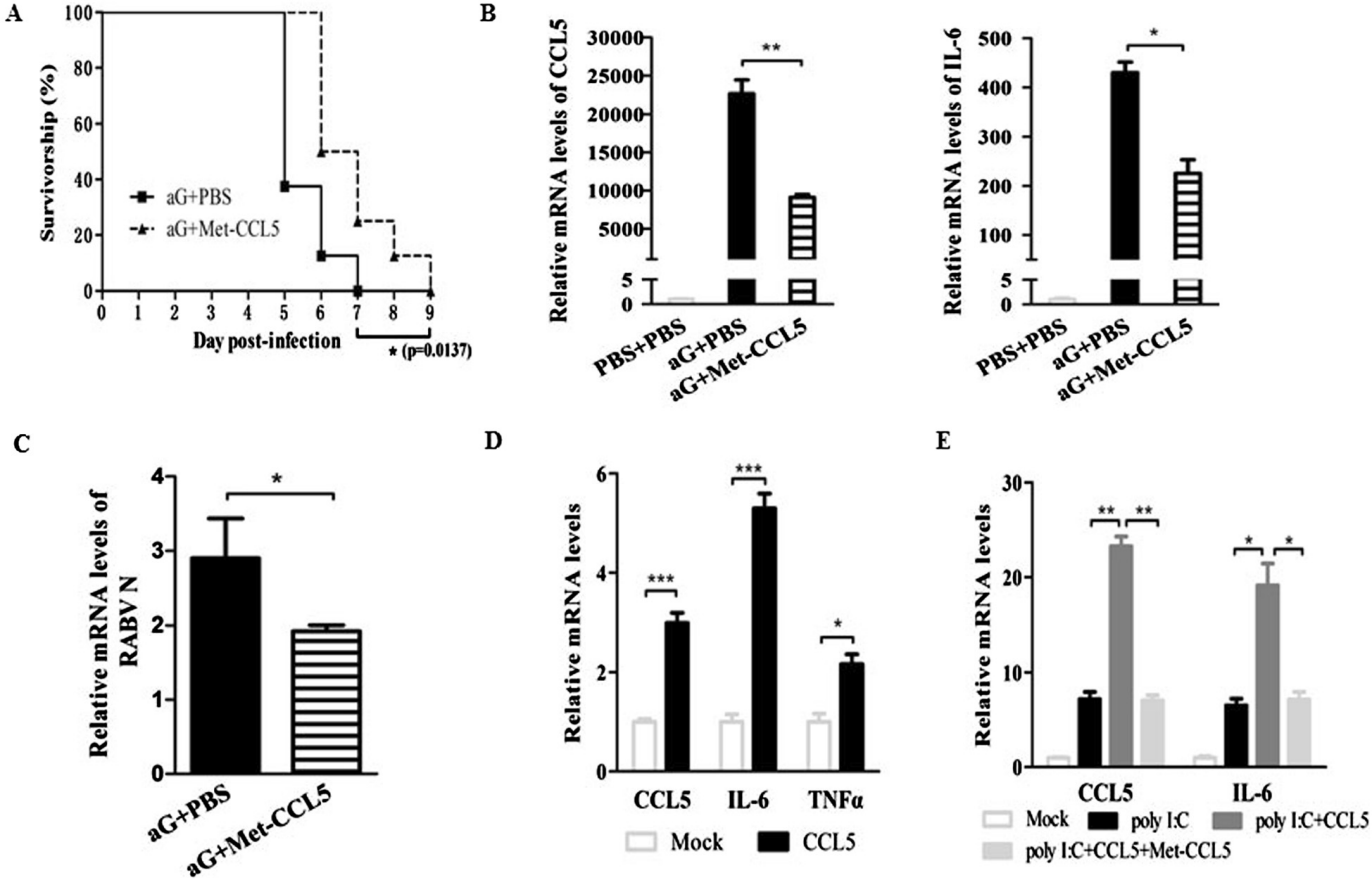
**Met-CCL5 treatment reduces the central nervous system inflammation after rabies virus infection.** Suckling mice were intracerebrally (i.c.) infected with aG (5.6 × 10^3^ FFU) followed by daily intraperitoneal (i.p.) administration with PBS or Met-CCL5 (100 μg/ml, 100 μl). **(A)** The survival rates in each group were recorded. **(B)** On day 5 postinfection (p.i.), the relative mRNA levels of CCL5 (**B**, left panel) and IL-6 (**B**, right panel) and RABV N protein **(C)** in the brains were quantified by quantitative reverse transcriptase PCR (qRT-PCR). **(D)** Bone marrow-derived macrophage (BMM) were treated with CCL5 (60 ng/ml, 6 hrs), and the relative mRNA levels of CCL5, IL-6 and TNF-α were measured by qRT-PCR. **(E)** BMMs were stimulated for 6 hrs with poly I:C alone, in the absence or presence of CCL5 or CCL5 and Met-CCL5. The relative mRNA levels of CCL5 and IL-6 were determined using qRT-PCR. Data were presented as means ± SD (*P <0.05; **P <0.01; ***P <0.001).

Met-CCL5, widely used as a CCL5 antagonist, could competitively bind to CCR5 and CCR1 to inhibit function and signaling pathways evoked by CCL5 [29,31]. Since RABV-induced CCL5 or exogenous administration of CCL5 could increase the numbers of immune cells infiltrating the CNS and enhance neuron apoptosis, there was an investigation as to whether *in vivo* administration of Met-CCL5 could lessen the severity of RABV infection. Met-CCL5 (10 μg/mouse) or PBS was i.p. injected daily into aG-infected suckling mice. Compared with PBS-treated mice, Met-CCL5 treated mice showed significantly prolonged survival time with an extra 2 days (*P* = 0.0137) (Figure 5A), and the mRNA levels of CCL5, IL-6 (Figure 5B) and RABV N protein (Figure 5C) were significantly downregulated in Met-CCL5 treated mice, suggesting that Met-CCL5 could reduce the production of pro-inflammatory cytokines and chemokines and prolong the survival time of attenuated RABV-infected suckling mice. To further assess how CCL5 and Met-CCL5 directly affect macrophages in regard to the production of CCL5 and pro-inflammatory cytokines, bone marrow-derived macrophages (BMMs) were treated with several stimuli *in vitro*. BMMs stimulated with CCL5 significantly increased the mRNA levels of CCL5, IL-6 and TNF-α (Figure 5D). It has been suggested that poly (I: C), a synthetic mimic of dsRNA, has been used extensively as a TLR3 ligand to mimic RNA virus infection and activate the TLR3 signaling pathway for subsequent expression of cytokines and chemokines, including CCL5 and IL-6 [40,41]. It was observed that CCL5 and IL-6 were upregulated in poly (I:C) treated BMMs and were further enhanced when BMMs were treated with poly (I:C) plus CCL5. Interestingly, Met-CCL5 treatment significantly reduced poly (I:C) and CCL5-induced CCL5 and IL-6 production in BMMs, which were comparable to those in poly (I:C)-stimulated BMMs (Figure 5E).

### Met-CCL5 treatment prolongs survival time of the mice infected with attenuated and street rabies viruses

**Figure 6:**
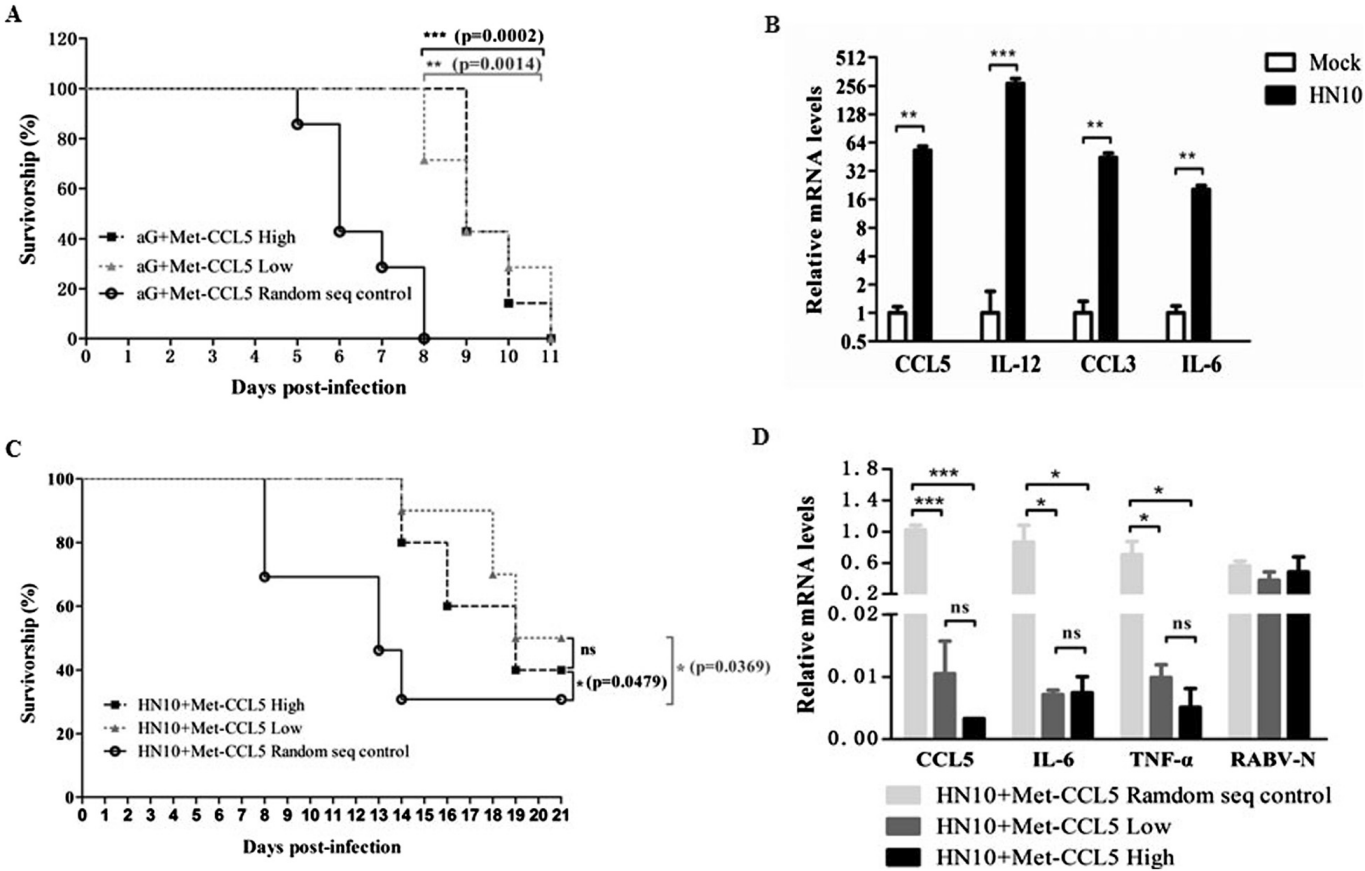
**Met-CCL5 treatment prolongs the survival time in adult mice after rabies virus infection.** The 6-week-old ICR mice (n ≥ 3) were intramuscularly (i.m.) infected with HN10 (5.6 × 10^3^ FFU), and the relative mRNA levels of CCL5, IL-12, CCL3, IL-6 were evaluated by quantitative reverse transcriptase PCR (qRT-PCR) **(B)**. The 6-week-old ICR mice were intracerebrally (i.c.) infected with aG or intramuscularly (i.m.) infected with HN10 followed by intraperitoneal (i.p.) administration of 100 μl Met-CCL5 at a concentration of 200 μg/ml or 20 μg/ml and 100 μl synthesized random disordered Met-CCL5 (200 μg/ml) as negative controls from day 2 postinfection (p.i.). Mice were monitored for 21 days, and the survival rate in the aG-infected group **(A)** and in the HN10 infected group **(C)** were recorded (n = 10; *P <0.05; **P <0.01; ***P <0.001). When the negative control mice infected with HN10 showed clinical signs, Met-CCL5 treated mice and control mice were euthanized at the same time, brains were removed, and these brains were subjected to analysis for pro-inflammatory chemokine/cytokine expression and virus load using qRT-PCR **(D)**. Data were presented as means ± SD (*P <0.05; **P <0.01; ***P <0.001).

To further assess whether Met-CCL5 treatment protected adult mice from aG infection *in vivo*, a control protein containing the same amino acids as Met-CCL5 with a random sequence was synthesized and used as a negative antagonist control. Adult mice were i.c. infected with aG, then i.p. treated with Met-CCL5 or negative antagonist daily from day 2 p.i.. All mice treated with the negative antagonist died within 8 days after infection with aG, whereas the mice treated with the high or low dose of Met-CCL5 exhibited prolonged survival time for an extra 3 days (*P* = 0.002 or *P* = 0.0014) (Figure 6A). No significant differences in survival time were observed between high and low doses of Met-CCL5-treated mice. Collectively, these data suggest that Met-CCL5 might compete with excessive CCL5 in the CNS to reduce inflammation and protect adult mice from attenuated RABV infection.

To mimic RABV infection in a natural route, adult mice were i.m. infected with street RABV strain HN10. First, we assessed whether excessive inflammatory cytokines and chemokines existed in the CNS of the HN10-infected adult mice. As shown in Figure 6B, the mRNA levels of CCL5, CCL3, IL-12 and IL-6 were significantly upregulated in the brain of the HN10-infected adult mice. Since Met-CCL5 could reduce inflammation and prolong survival time in the aG-infected suckling and adult mice, we suspect and further investigate whether Met-CCL5 treatment could also protect adult mice from the street RABV infection via an i.m. route. Adult mice were i.m. infected with HN10 and then injected with Met-CCL5 daily i.p. from day 2 p.i. (high dose: 20 μg/mouse or low dose: 2 μg/mouse). As shown in Figure 6C, Met-CCL5 significantly prolonged the survival time of HN10-infected adult mice compared to the control mice. In the early days of infection (that is, day 0 to day 14), Met-CCL5 treatment substantially increased survival rates of these infected mice compared to control protein-treated mice (that is, 80% versus 30% survival rates at day 14). Similar to the effect observed in the aG-infected suckling mice, there was no significant difference between high dose and low dose of Met-CCL5 treated groups. Importantly, the mRNA levels of CCL5, IL-6 and TNF-α were significantly reduced in the CNS of Met-CCL5 treated mice compared to the CNS in control mice (Figure 6D), and no significantly reduced virus load was detected in Met-CCL5 treated mice. Despite the protective role of Met-CCL5 in the early days of infection, over 50% of Met-CCL5 treated adult mice still died after 19 days p.i. (Figure 6C), suggesting that additional treatment might be combined with Met-CCL5 to improve anti-RABV efficacy.

## Discussion

RABV is a highly neurotropic virus inducing acute infection in the CNS. Chemokines are now recognized as critical regulators of leukocyte trafficking in the CNS, and numerous studies have revealed that resident cell populations of the CNS are able to synthesize and secrete a variety of chemokines. Neurons, microglia and astrocytes are the primary sources of chemokines following infection with a wide range of neurotropic viruses, including RABV [42], WNV [43], and herpes simplex virus 1 (HSV1) [44]. *In vitro* studies have highlighted that infection of neurons with RABV results in robust production of chemokines [42]. In this study, we screened the expression levels of 40 inflammatory molecules in the CNS and demonstrated that CCL5 is the most significantly upregulated chemokine in response to aG and CTN infection in suckling mice, which suggested chemokine CCL5 is one key immune regulator during RABV infection.

Chemokines are critical mediators of neuropathology during viral infections in the CNS, either by attracting pathogenic inflammatory cells or directly mediating neurotoxicity and cell death. Chemokines regulate infiltration of immune cells to the CNS after viral infection by enhancing blood-brain barrier (BBB) permeability [45]. These immune cells release additional chemokines, such as CCL5, to further recruit macrophages, monocytes and T cells, consequently increasing the severity of demyelination [23]. A previous study showed that recombinant RABV expressing CCL5 or IP-10 increased pathogenicity with excessive infiltration of inflammatory cells into the CNS [11]. In this study, excessive infiltration of T cells, macrophages or activated microglia and neuron apoptosis was detected in mice infected with attenuated RABVs, which is in agreement with what others have reported [5]. Moreover, our *in vivo* and *in vitro* data suggested that CCL5 played an essential role in enhancing trafficking of immune cells in the CNS during RABV infection.

CCL5 belongs to the CC family of chemokines and plays an important role in recruiting T cells, macrophages and monocytes to the site of inflammation by interacting with the specific G protein-coupled receptors CCR1, CCR3 and CCR5 [20,21]. The binding of CCL5 and its receptors CCR5 activates a series of downstream effectors that facilitate leukocyte trafficking into the CNS [37]. Although immune cell infiltration and anti-viral activity is requisite for viral clearance, excessive accumulation of leukocytes within the CNS results in neuropathology [11]. Met-CCL5 is a CCL5 receptor antagonist, which is used extensively to block the pathogenic effects of CCL5 and attenuate CCR5-mediated inflammatory processes during the development of arthritis, colitis, airway inflammation, allograft rejection and chronic liver diseases [46-50]. Moreover, CCR5 is involved in HIV entry to target cells, so CCR5 antagonists have been successfully tested in phase III studies in patients with HIV infection. In this study, i.p. administration of CCL5 antagonist Met-CCL5 significantly reduced CCL5 and IL-6 in aG-infected suckling mice. Other studies have shown that street RABVs also induce a strong inflammatory response in adult mice or dogs at the late stage of the infection [16,18]. Since upregulated inflammatory cytokines and chemokines were also observed in adult mice infected with street RABV by i.m. route, an investigation as to whether Met-CCL5 was protective in these mice was carried out. Indeed, Met-CCL5 significantly protected adult mice from the infection of the street strain HN10, especially at the early stage of infection. According to previous studies, pretreatment with Met-CCL5 at doses of 0.1 and 1 μg/mouse significantly inhibits cellular recruitment in a murine model of airway inflammation [49]; furthermore, administration of 10 μg Met-CCL5 for 3 days (30 μg in total) at the peak of liver fibrosis significantly inhibits fibrosis progression and accelerates its regression in chronic liver diseases [51]. In our study, administration of low dose Met-CCL5 (2 μg daily; 20 μg for aG-infected or 30 μg for HN10-infected adult mice in total) was enough to inhibit RABV infection. This might explain why no significant difference was observed between low (2 μg) and high (20 μg) doses of Met-CCL5 treatment.

However, a limited protective role of Met-CCL5 was observed at the late stage of infection. Considering the complex pathogenic reasons for the lethal RABV infection, no single therapeutic reagent is likely to be effective. Therefore, it is crucial to consider a combination of Met-CCL5 therapy with other treatments to improve protection against RABV. Efforts aimed at developing successful therapeutics to combat RABV infection have been in progress for decades. Despite attempts at drug development and antiviral regimens, there are still no effective treatments for use in the clinic. Several reports have suggested that RABV neutralizing monoclonal antibody cocktails could protect mice from a lethal dose of RABV infection [52,53]. Therefore, if Met-CCL5 treatment could open a therapeutic window at the early stage, it would be fundamental to further investigate whether Met-CCL5 and the RABV neutralizing monoclonal antibody cocktails could be used as a combined therapy to protect the host from RABV infection.

## Abbreviations

Abs: antibodies
BBB: blood-brain barrier
BILs: brain infiltration of lymphocytes
BMM: bone marrow-derived macrophages
CNS: central nervous system
FFU: fluorescent focus forming unit
i.c.: intracerebrally
i.p.: intraperitoneally
p.i.: postinfection
qRT-PCR: quantitative reverse transcriptase PCR
PI: propidium iodide
RABV: rabies virus
SDS-PAGE: sodium dodecyl sulfate polyacrylamide gel electrophoresis
TUNEL: terminal deoxynucleotidyl transferase dUTP nick end labeling
VNA: virus neutralizing antibody
